# Prevalence of markers of beta cell autoimmunity and thyroid disease in children with coeliac disease

**DOI:** 10.1186/s12887-023-04294-6

**Published:** 2023-09-16

**Authors:** Corinne Légeret, Alexander Kutz, Brunner Jessica, Esther Mundwiler, Henrik Köhler, Luca Bernasconi

**Affiliations:** 1 Medical Faculty, University Children’s Hospital Basel, Spitalstrasse 33, Basel, 4056 Switzerland; 2 Division of Pharmacoepidemioloy and Pharmacoeconomics, Department of Medicine, Brigham and Women’s Hospital, Harvard Medical School, 1620 Tremont Street, Suite 3030, Boston, 02120 MA USA; 3 Medical University Department, Division of General Internal and Emergency Medicine, Cantonal Hospital Aarau, Aarau, Switzerland; 4 Institute of Laboratory Medicine, Kantonsspital Aarau AG, Tellstrasse 25, Aarau, 5001 Switzerland; 5 Children’s Hospital Aarau, Kantonsspital Aarau AG, Tellstrasse 25, Aarau, 5001 Switzerland

## Abstract

**Background:**

Over the last decades, the prevalence of coeliac disease (CD), an autoimmune disorder, rose to 1–2%. Whether patients with CD have higher risk of developing other autoimmune disorders such as type 1 diabetes, Hashimoto thyroiditis, or Graves` disease remains unclear.

**Aim:**

The aim of this study was to determine the prevalence of biomarkers of beta cell and thyroid autoimmunity in children with CD.

**Methods:**

Retrospective cross-sectional cohort study comparing pediatric patients suffering from CD with age and sex-matched healthy controls (HC). Participant`s serum was tested by immunoassay for following autoantibodies (aAb): TSH-receptor antibodies (TRAb), anti-thyroglobulin (anti-Tg), anti-thyroid peroxidase (anti-TPO), anti-glutamic acid decarboxylase (anti-GAD), anti-zinc transporter 8 (anti-ZnT8), anti-islet antigen 2 (anti-IA2) and anti-insulin.

**Results:**

A total of 95 patients with CD (mean age 8.9 years; 63% female) and 199 matched healthy controls (mean age 9.2 years; 59.8% female) were included in the study. For patients with CD, a seroprevalence of 2.1% (vs. 1.5% in HC) was calculated for anti-GAD, 1.1% for anti-IA2 (vs. 1.5% in HC), 3.2% for anti-ZnT8 (vs. 4.2% in HC), and 1.1% (vs. 1% in HC) for anti-insulin. For thyroid disease, a seroprevalence of 2.2% for TRAb (vs. 1% in HC), 0% for anti-TPO (vs. 2.5% in HC) and 4.3% for anti-Tg (vs. 3.5% in HC) was found for patients with CD.

**Conclusion:**

This study suggests a higher prevalence of autoimmune antibodies againstthyroid in children with CD compared to HC, whilst it is similar for pancreatic antibodies. Prospective cohort studies are needed to first evaluate the occurrence of autoimmune antibodies against beta cells and thyroid over a longer follow-up time and second to explore their clinical relevance.

## What is known

• The prevalence of coeliac disease in children is on the rise and it is markedly overrepresented in children suffering from type I diabetes mellitus (T1DM).

• Recommendations for how/when to screen children with T1DM for coeliac disease are available, but there is only sparse data about children with coeliac disease developing T1DM or thyroidal disease.

## What is new

• This study confirms a higher prevalence of autoimmune antibodies against thyroid in children with CD compared to healthy control.

• Although a statistically significant correlation between anti-tissue-transglutaminase IgA and anti-thyroglobulin and TSH receptor antibodies was shown, the clinical significance (development of possible disease) of it remains unclear due to the cross-sectional study design. The same circumstances apply to T1DM antibodies.

## Background

Coeliac disease (CD) was long considered to be a rare disease occurring only in young children with the lead symptom of malabsorption. Over the last decades, prevalence of children and teenagers suffering from CD rose to 1–2%, becoming one of the most common lifelong autoimmune disorders with more patients presenting with less acute clinical manifestation [1]. Nowadays it is clear, that CD is a complex immune-mediated systemic disorder triggered by gluten in genetically predisposed patients [2]. In affected patients, the consumption of gluten leads to the activation of CD4 + T cells, which causes the release of pro-inflammatory cytokines and chemokines, favoring inflammatory cell infiltration and CD8 + T cell cytotoxic activity and contributes to mucosal damage [3]. Although in the last decades a deeper insight in the autoimmune pathophysiology has been gained, CD still seems to be largely underdiagnosed, as a high proportion of children suffer from a clinically silent course (microscopically mucosal damage is detectable in asymptomatic children) and a persistence of insufficient awareness of the variability of the disease amongst clinicians [4]. The reported prevalence is clearly lower in countries where children with known risk factors are not actively screened for CD, mainly due to limited health care access or resources [5, 6]. Previously, the diagnosis was based on histopathologic results from the small bowel biopsy, but since 2012 the diagnostic algorithm has evolved owing to the availability of specific serological biomarkers [7].

Children with a selective IgA deficiency [8], a Down syndrome [9], Turner syndrome [10] or Williams syndrome [11] have a higher risk for CD, therefore the indication for serological screening of these groups is given. There is not only a significant association with selected syndromes, but also patients with other autoimmune diseases, such as type 1 diabetes (T1DM), autoimmune hepatitis, primary biliary cholangitis, systemic lupus erythematosus, inflammatory bowel disease, and systemic sclerosis [12–15] are more often diagnosed with CD than the normal population.

CD is markedly overrepresented in patients suffering from T1DM: studies suggest an incidence of CD in patients with T1DM ranging from 5 to 10% [16], thus it is recommended to screen those patients for CD annually [17]. For the opposite situation, however, i.e. children and adolescents with CD, there are almost no data regarding the development of diabetes or thyroid disease, therefore no recommendations exist. To further assess the potential interplay between CD and the prevalence of beta cell autoimmunity or autoimmune thyroid diseases, we aimed to test different autoimmune antibodies against beta cells and thyroid in children with CD and matched healthy controls (HC).

## Methods

This was a retrospective cross-sectional cohort study to test four markers of beta cell autoimmunity and three thyroid autoantibodies in children with confirmed CD and in age and sex-matched healthy controls.

Since July 2017, residual serum samples of pediatric patients screened for celiac disease by the measurement of anti-tissue-transglutaminase (anti-tTG IgA) were stored in the biobank of the Children’s Hospital Aarau, Switzerland. Children and adolescents between the age of 0 and 18 years, who were diagnosed with CD according to the European guidelines [6] and had a positive anti-tTG IgA, were included in the study. In order to compare the results with a healthy population, age and sex-matched children and adolescents with negative anti-tTG IgA and a diagnosis of functional abdominal disease were enrolled. Following information was obtained from all patients’ medical records: date of birth, sex, date and results of anti-tTG IgA, family history, comorbidities, main symptom why CD screening was performed, weight and height with corresponding z-score (Swiss national percentiles were used [18]).

We defined the following inclusion criteria for CD patients: age between 0–19 years, residual serum in which a positive t-transglutaminase has been tested and a confirmed diagnosis of CD according to the European guidelines [6]. As CD patients have repeatedly their blood tested, especially in the beginning after making the diagnosis, we chose the blood sample of each patient with the highest value for the t-Transglutaminase, and it always had to be positive. Therefore, the age when the diagnosis was made does not necessarily correspond to the age when the blood sample was taken with the highest t-transglutaminase (corresponding to ‘mean age at enrollment’ in Table 1). We wanted the t-transglutaminase to be as high as possible, as this may also trigger other autoimmune antibodies. Exclusion criteria for healthy control were positive t-Transglutaminase or an underlying diagnosis of CD. Any other autoimmune disease (e.g. juvenile arthritis, thyroiditis) were exclusion criteria for all participants.

**Table 1:** Patient’s characteristics

	Patients with coeliac disease			Healthy controls		
	Male (n= 35)	Female (n=60)	Total (n=95)	Male (n=80)	Female (n=119)	Total (n=199)
Mean age at diagnosis in years (range)	**7.1** (1.25 – 15.25)	**8.7** (2-17)	**8.1** (1.25-17)			
Mean age at enrollment (range)	**8.2** (2.0 16.5)	**8.9** (2-18.25)	**8.9** (1.5 – 18.25)	**9.2** (1.5 – 16.5)	**9.2** (2-19)	**9.2** (1.5 -19)
Mean z-score for weight (range)	**-0.2** (-2.5 – 2.5)	**-0.4** (-2.6 – 2.5)	-**0.3** (-2.6 – 2.6)	**-0.1** (-2.6 – 3)	**-0.2** (-3 – 3)	**-0.2** (-3 – 3)
Mean z-score for length (range)	**-0.2** (-2.6 – 2.4)	**-0.4** (-3.3 – 1.8)	**-0.3** (-3.3 – 2.4)	**-0.5** (-3.8 – 2.8)	**0.2** (-2.8 – 2.7)	**-0.1** (-3.8 – 2.8)
**Leading symptom, why patient was tested for coeliac disease n (%)**						
abdominal pain		32 (33.7%)		54 (27%)		
failure to thrive/ growth below genetic centile		21 (22.1%)		51 (25.6%)		
constipation		5 (5.3%)		42 (21.1%)		
flatulence		1 (1%)		2 (1%)		
fatigue		3 (3.2%)		2 (1%)		
iron deficiency/anemia		6 (6.3%)		6 (3%)		
screening due to pos. family history		9 (9.4%)		-		
screening due to trisomy 21		2 (2.1%)		-		
vomiting		-		5 (2.5%)		
diarrhea		6 (6.3%)		2 (1%)		
others		10 (10.5%) dysphagia, oral ulcer, nausea, urticaria, low appetite, elevated liver parameters, soft stool, vaginal itchiness		35 (17.6%) dysphagia, anorexia, gastroesophageal reflux, arthritis, amenorrhea, cystitis, eczema, nausea, elevated liver parameters		

1c) TRAb in U/ml 1d) anti-TPO in U/l

### Laboratory measurements

Anti-tTG IgA (EliA, Thermo Fisher Scientific, Sweden), IgA and TSH (Siemens Healthyneers, Germany) were routinely performed. Autoantibodies (aAb) were measured as follow: Anti-TPO, anti-Tg and TRAb (EliA, Thermo Fisher Scientific, Sweden); anti-GAD, anti-IA2 and anti-ZnT8 (ELISA, RSR, UK); anti-insulin (ELISA, AESKU Diagnostics, Germany).

For the semi-quantitative interpretation of the aAb results the following criteria were used: anti-GAD (U/ml): < 5 negative; 5–9: +; 10–49: ++; ≥50: +++. Anti-ZnT8 (U/ml): <15 negative; 15–29: +; 30–149: ++; ≥150: +++. Anti-IA2 (U/ml): < 15: negative; 15–29: +; 30–149: ++; ≥150: +++. Anti-Insulin (U/ml): < 18: negative; 18–35: +; 36–179: ++; ≥180: +++. A (U/l): < 3.4: negative; 3.4–3.9: +; 4-5.9: ++; ≥ 6: +++. Anti-TPO (U/ml): ≤ 35: negative; 36–99: +; 100–499: ++; ≥500: +++. Anti-TG (U/l): ≤ 601: negative; 61–99: +; 100–499: ++; ≥ 500: +++.

### Statistical analysis

Categorical variables are expressed as numbers (percentage) and continuous variables as means (± SD) or medians (interquartile range (IQR)) as appropriate. The relationship between different antibodies was investigated by using a spearman’s rank correlation analysis and reported as spearman’s rank coefficient rho with the corresponding p-value.

Data for antibodies have been log-transformed to achieve nearly normal distribution. We used simple linear regression technique adjusting for left-censoring to assess differences in antibody levels between the two groups. All p-values are two-sided, all confidence intervals are at the 95% level. Statistical analyses were performed at an alpha-level of 5% using Stata, version 17.1 (StataCorp LLC), MEDCALC (MedCalc Statistical Software version 19.3; MedCalc Software bv, Ostend, Belgium) and Excel.

### Ethical statement

The study was conducted in accordance to the ethical principles laid down in the declaration of Helsinki and its later amendments. Furthermore, it was approved by the local ethical committee (Ethics committee of Northwest Switzerland, EKNZ, trial number 2019 − 00869).

## Results

Among 320 eligible subjects, 16 CD patients and one healthy control did not have enough sample volume left to complete laboratory measurements and were therefore excluded from the study, as well as 9 patients with an underlying autoimmune diseaseThus, a total of 95 patients with CD (mean age 8.9 years; 63% female) and 199 HC (mean age 9.2 years; 59.8% female) were enrolled. Weight and length distribution was similar in both groups. The leading symptom to screen patients for CD was overall abdominal pain, followed by failure to thrive/growth below genetic centile (Table 1).

### Seroprevalence

Of 95 patients with CD, the seroprevalence for anti-GAD was 2.1% (vs. 1.5% in HC), 1.1% for anti-IA2 (vs. 1.5% in HC), 3.2% for anti-ZnT8 (vs. 4.2% in HC), and 1.1% (vs. 1% in HC) for anti-insulin.

For thyroid disease, a seroprevalence of 2.2% was found for TRAb (vs. 1% in HC), 0% for anti-TPO (vs. 2.5% in HC) and 4.3% for anti-Tg (vs. 3.5% in HC), see Table 2.

**Table 2:** Semi-quantitative classification of pancreatic and thyroid antibodies results

Pancreatic antibodies		Seroprevalence	Seronegative	+	++	+++
**Anti-GAD**	CD n= 93	2 (2.1%)	91 (97.8%)	1	0	1
	HC n= 191	3 (1.5%)	188 (98.5%)	0	3	0
**Anti-ZnT8**	CD n= 92	3 (3.2%)	89 (96.7%)	1	0	2
	HC n= 190	8 (4.2%)	182 (95.8%)	6	1	1
**Anti-IA2**	CD n=92	1 (1.1%)	91 (98.9%)	0	0	1
	HC n= 190	3 (1.5%)	187 (98.5%)	2	1	0
**Anti-Insulin**	CD n=92	1 (1.1%)	91 (98.9%)	0	1	0
	HC n=195	2 (1%)	193 (98.9%)	2	0	0
**All T1DM antibodies**	CD n= 369	7 (1.9%)	362 (98.1%)	2	1	4
	HC n=766	18 (2.3%)	748 (97.6%)	12	1	1
**Thyroid antibodies**		**Seroprevalence**	**Seronegative**	**+**	**++**	**+++**
**TRAb**	CD n=93	2 (2.2%)	91 (97.8%)	0	1	1
	HC n=199	2 (1%)	197 (99%)	2	0	0
**Anti-TPO**	CD n=93	0 (0%)	103 (99%)	0	0	0
	HC n=198	5 (2.5%)	193 (97.5%)	3	2	0
**Anti-Tg**	CD n= 93	4 (4.3%)	89 (95.7%)	1	3	0
	HC n= 199	7 (3.5%)	192 (96.5%)	3	1	3
**All thyroid antibodies**	CD n=279	6 (2.1%)	273 (97.8%)	1	4	1
	HC n=596	14 (2.3%)	582 (97.7%)	8	3	3

HC (healthy controls); CD (coeliac disease)

### Association of CD, antibody levels and TSH

**Fig. 1:**
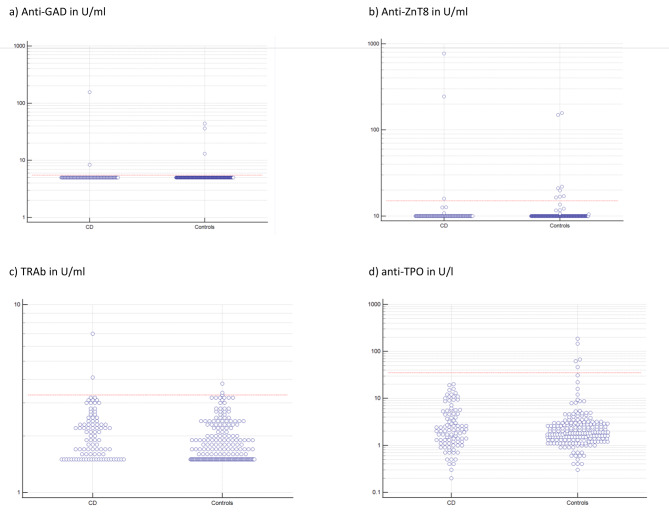
Graphs of auto- antibodies in patients with coeliac disease (CD) and healthy controls

Except for anti-Tg, Anti-TPO and anti-GAD, measured circulating antibody levels were higher in patients with CD as compared with their healthy controls (see Fig. 1). Similar, the mean level of TSH was also higher in CD patients. Among biomarkers of beta cell autoimmunity, mean levels of Anti-ZnT8, anti-insulin and Anti-IA2 were higher in patients with CD with a relative between-group difference of 1.76 to 4.04%. Among anti-thyroid antibodies, mean TRAb level was higher in CD patients with a between-group difference of 9.5% (Table 3).

**Table 3:** Thyroid disease and diabetes mellitus screening measures among patients with and without celiac disease

	Healthy controls (n = 200)	Mean of positive antibodies in healthy controls	Celiac disease (n = 95)	Mean of positive antibodies in patients with celiac disease	Relative between-group difference, % (95% CI)	Spearman’s rank correlation between t-Transglutaminase & antibodies
**Thyroid disease, mean (SD)**						
TSH, mU/l	1.79 (1.66)		1.90 (1.92)		6.16 (-8.94 to 22.78)	0.085; p = 0.23
TRAb, U/l	1.83 (1.27)	3.6	2.00 (1.33)	5.6	9.50 (2.86 to 16.56)	0.255; p<0.001
Anti-Tg, U/l	10.02 (2.34)	621	8.99 (2.24)	174	-10.26 (-27.04 to 10.39)	-0.163; p = 0.01
Anti-TPO, U/l	2.17 (2.45)	185	2.32 (2.74)	-	6.69 (-15.29 to 34.39)	-0.036; p = 0.59
**Diabetes mellitus, mean (SD)**						
Anti-GAD, U/ml	5.14 (1.25)	205.2	5.22 (1.43)	82.2	1.60 (-5.13 to 8.80)	0.026; p = 0.71
Anti-ZnT8, U/ml	10.55 (1.35)	52.5	10.97 (1.75)	343	4.04 (-5.95 to 15.08)	-0.023; p = 0.74
Anti-IA2, U/ml	15.29 (1.19)	62.4	15.56 (1.42)	432	1.76 (-4.34 to 8.24)	0.075; p = 0.276
Anti-insulin, U/ml	3.41 (1.32)	17.9	3.38 (1.41)	56	-0.75 (-7.92 to 6.98)	-0.020 ; p = 0.76

TSH (thyroid stimulating hormone), TRAb (TSH receptor antibodies), anti-Tg (thyroglobulin antibodies), anti-TPO (thyroid peroxidase antibodies), anti-GAD (glutamic acid decarboxylase antibodies), ZnT8 (zinc transporter 8 antibodies), anti-IA2 (insulinoma antigen2 antibodies), CI (confidence interval)

### Correlation of t-Transglutaminase, antibody levels and TSH

While there was no evidence for correlation between anti-tTG IgA and markers of beta cell autoimmunity, it was weakly correlated with TRAb (rho = 0.26, p < 0.001) and anti-Tg (rho=-0.16, p = 0.01), respectively.

## Discussion

This observational study investigated the association of CD and different antibodies against beta cells and thyroid and revealed two key findings: First, most antibody levels were numerically higher in patients with CD as compared with their matched controls. Secondly, in a quantitative analysis, there was evidence that mean levels of Anti-insulin, Anti-ZnT8, Anti-IA2, and TRAb were higher in patients with CD.

It is well known that patients with a diagnosis of T1DM develop significantly more often CD than healthy controls [19]. Previous studies showed prevalence rates of 3.7% in Israel [20], 4.8% in Greece [21], 6.4% in Germany [22], and 11.1% in India [23] with an overall female dominance. Therefore, it is widely recommended to annually screen people with T1DM for potential co-presence of CD [16, 24]. A shared genetic background of T1DM and CD seems likely. While HLA class II genes as DQ 2 and 8 are present in 95% of patients with T1DM and in around 99% of CD patients [16], this is the case in only 40% of the unaffected population [25]. However, genetics cannot exclusively explain this occurrence as the rapid increase in incidences for both diseases points to environmental changes [26]. Although a certain degree of similarity is given, only sparse literature is available for the presence of markers of beta cell autoimmunity or autoimmune thyroid disease in patients with CD. A previous study in adults showed a prevalence of 3.8% for T1DM in patients with CD [27], with a strong predominance for male (8% vs. 1.8% females) and younger patients. In our cohort of pediatric patients with CD we recorded an overall prevalence of 0% for T1DM and mean variables for ¾ of beta cell autoantibodies were higher than in the age and sex-matched controls. The largest between-group differences were observed for anti-ZnT8 and anti-IA2. T1DM can be diagnosed based on the identification of antibodies at pre-symptomatic stages [28], which can be subdivided into two stages. Stage 1 is defined as the presence of two or more autoantibodies (aAb) to insulin, IA-2, GAD or ZnT8 whilst the blood glucose levels remain within normal range [29]. At stage 2, patients are still asymptomatic but due to β cell loss blood sugar levels become abnormal and aAb are still detectable. Antibodies against ZnT8A are present in 60–80% of patients at the beginning of the disease and can be detected in 26% of patients, in whom no GAD or IA-2 antibodies are detected, having a high prevalence in children [30, 31]. To date ZnT8 aAb seems to identify patients at higher risk to develop T1DM at the earliest possible stage [32], which is in line with the finding of our cohort of patients with a higher risk to develop T1DM and a tendency for higher values. However, one of our patients with CD did have an isolated positive ZnT8 aAb, without a diagnosis of T1DM. Literature confirms a higher prevalence of ZnT8 positive patients between 8 and 19 years [33, 34] at the very beginning of the autoimmune process. The mean for anti-ZnT8 in our patients with CD were 6 times above that of HC, the Spearman’s rank correlation with anti-tTG IgA was not statistically significant. Before anti-ZnT8 were available, initial positivity for anti-GAD was considered to be a risk factor for a development of an autoimmune diabetes, as shown in a cohort of over 6000 pediatric patients (TEDDY study, 36): anti-GAD, anti-IA-2 and anti-insulin were analyzed from 3 months onwards up to 15 years of age quarterly in pediatric patients with a genetic risk for T1DM. The first aAb to become positive before onset of T1DM was GAD and/or IA-2. In 2020, a follow-up of the TEDDY study [36] was published, showing that most patients firstly had positive values for GAD and a younger initial age at seroconversion and shorter time to the development of the second-appearing aAb increased the risk for T1DM. In our cohort, the seroprevalence of anti-GAD was slightly higher for children with CD.

A large Swedish inpatient register study of over 9’000 children with CD found a statistically significant increased risk of subsequent T1DM and of a ketoacidosis or diabetic coma before the age of 20 years [37]. However, data on more specific risks (e.g. aAb constellation etc.) for CD patients does not exist, therefore no recommendations are available whether to screen CD patients for T1DM. A literature search showed that screening for celiac disease is not indicated in children with the autoimmune rheumatologic diseases lupus erythematosus and juvenile idiopathic arthritis, as no increased incidence was shown in affected individuals [38, 39].

Autoimmune thyroid diseases include Hashimoto thyroiditis (HT) and Graves-Basedow disease in the pediatric population. Hashimoto thyroiditis represents the most common cause of acquired hypothyroidism in geographic areas lacking iodine, whilst GD is the most common disease leading to hyperthyroidism in children and adolescents [40]. Hashimoto’s thyroiditis is defined by the presence of high serum thyroid antibody concentrations (anti-Tg and/or anti-TPO) accompanied by hypothyroidism or goiter [41]. For patients with CD, a prevalence of 2–10%, therefore 3–4 times higher than in general population, was found for autoimmune thyreopathies [42, 43]. A Turkish study group found 3% of their 66 paediatric patients with autoimmune thyroiditis to have CD [44]In our cohort of patients with CD we found a higher seroprevalence of anti-Tg and TRAb compared to the age-and sex-matched controls and the Spearman’s rank correlation between the anti-tTg-IgA and those two antibodies was statistically significant.

A previous study exploring the association of CD and anti-thyroid antibodies in patients with T1DM found a lower prevalence in the patients with CD than in diabetic non-CD patients (13% vs. 19%) [44]. It was speculated whether the presence of T1DM could be a stronger risk factor for the presence of anti-thyroid antibodies than CD per se.

A study assessing adult CD patients [45], a prevalence of 21% was found for a positive thyroid serology (anti-Tg and/or anti-TPO). Of them, 90% patients were positive for anti-TPO and only in 46% they had a positive anti-Tg. Only 12% were diagnosed with Hashimoto thyroiditis, whereas the remaining had normal thyroid biochemistry. As the values for TSH were within normal range for our patients, no diagnosis of a manifest Hashimoto thyroiditis was made until this manuscript was finished. It is to mention, that Rodriguez et al. [46] found positive results for anti-TPO and anti-TG in 15% of the general population without any symptoms.

The literature regarding the prevalence of autoimmune thyroid disease in children with CD varies: Ventura et al. found a prevalence of 14.4% [47], whereas a Turkish study 2–3 years into the diagnosis of CD found a prevalence of 16%, whilst none of the included 67 patients had detectable aAb against the thyroid at diagnosis [48]. In contrast, Wessels et al. [49] found no benefit in screening children with CD for thyroidal disease, therefore data in the literature remains controversial. We observed an overall seroprevalence of 2.1% (vs. 2.3% in HC) for antithyroid aAb, but on average the diagnosis of CD was only made 0.8 years before the serum was analyzed. Based on the study above, it is assumed that it does take time from making the diagnosis of CD until a seroconversion can be observed but the duration remains unclear. There clearly seems to be a correlation between patients with CD and thyroidal abnormalities, but a discrepancy in different data persist, which is consistent with the current European Guidelines for the follow-up of paediatric patients with CD stating that ‘screening for thyroid disease with TSH and thyroxine may be considered’ [50].

Our study has limitations. First, the follow-up of this study was quite short, not allowing to observe a potential increase of antibody positivity and clinical development over time. Second, the sample size was small to detect clear associations, correlation and the external validity was not strong.

## Conclusion

This study confirms a higher prevalence of autoimmune antibodies against pancreas and thyroid in children with CD compared to healthy control with a statistically significant correlation between anti-tTG IgA and TRAb/anti-Tg.Although a statistically significant correlation between anti-tTG IgA and anti-Tg/TRAbwas shown, the clinical significance (development of possible disease) of it remains unclear due to the cross-sectional study design. The same circumstances apply to DM1-aAb. But based on the current literature, there is no evidence to advise whether aAb against thyroid and pancreas should be monitored and in which frequency. Prospective cohort studies are needed to shed light on the interplay of the different autoimmune diseases in paediatric patients with CD and evaluate the occurrence of aAb over the course of time with their predictive value.

## Data Availability

The dataset used is available from the corresponding author on reasonable request.

## Abbreviations

aAb: Autoantibodies
CD: Coeliac disease
CI: Confidence interval
GAD: Glutamic acid decarboxylase
HC: Healthy control
IgA: Immunoglobulin A
IA2: Islet antigen 2
T1DM: Type 1 diabetes mellitus
TPO: Thyroid peroxidase
Tg: Thyroglobulin
TRAb: TSH-receptor antibodies
TSH: Thyroid-stimulating hormone
tTG: Tissue Transglutaminase
SD: Standard deviation
ZnT8: Zinc transporter 8
